# Bone Marrow Mesenchymal Stem Cell‐Derived Exosomal miR‐451a Inhibits Macrophage M1 Polarization Through the TLR4/PI3K/AKT Signaling Pathway to Alleviate Acute Lung Injury

**DOI:** 10.1155/sci/5080421

**Published:** 2026-10-26

**Authors:** Jiawu Yang, Yongze Liu, Qinglang Dai, Yuan Liao, Liang Zhao, Zhu Tian, Feng Li

**Affiliations:** ^1^ Department of Pulmonary and Critical Care Medicine Kunming Children’s Hospital (Children’s Hospital Affiliated to Kunming Medical University) Kunming 650100 Yunnan China kmmc.cn

**Keywords:** acute lung injury, BMSCs, exosomes, macrophage polarization, miR-451a

## Abstract

**Background:**

Abnormal polarization of macrophages toward the M1 phenotype is a key pathogenic mechanism in acute lung injury (ALI). Bone marrow mesenchymal stem cell (MSC)‐derived exosomes (BMSC‐Exos) represent a promising therapeutic approach to inhibit macrophage M1 polarization and alleviate ALI, but the molecular mediators involved are incompletely defined. This study aimed to identify the responsible exosomal mediator and to define its downstream signaling.

**Methods:**

An ALI mouse model was established via intratracheal LPS instillation, and a cell injury model was constructed using LPS‐stimulated RAW264.7 cells. Exos were isolated from human BMSCs. Lung injury was assessed through HE staining, lung wet/dry weight ratio, bronchoalveolar lavage fluid (BALF) protein concentration, and total BALF cell count. The expression levels of related genes and proteins were assessed using RT‐qPCR, western blotting, ELISA, and immunofluorescence.

**Results:**

Treatment with BMSC‐Exos significantly ameliorated lung damage in ALI mice and reduced the lung wet/dry weight ratio, BALF protein concentration, and total inflammatory cell count. Additionally, BMSC‐Exos reduced the expression of M1 macrophage markers (iNOS, CD80, and CD86) and proinflammatory cytokines (IL‐1β, IL‐6, and TNF‐α), thereby inhibiting macrophage M1 polarization. Mechanistically, BMSC‐Exos suppressed the LPS‐induced activation of the PI3K/AKT signaling pathway, whereas the PI3K/AKT activator 740 Y‐P weakened the inhibitory effect of BMSC‐Exos on M1 polarization. The expression of miR‐451a was significantly downregulated in ALI and enriched in BMSC‐Exos. miR‐451a inhibits the PI3K/AKT signaling pathway by targeting and suppressing TLR4 expression. miR‐451a knockdown or TLR4 overexpression attenuated the therapeutic effects of BMSC‐Exos on ALI.

**Conclusion:**

BMSC‐Exos‐derived miR‐451a inhibited the PI3K/AKT signaling pathway by downregulating TLR4 expression, thereby suppressing macrophage M1 polarization and alleviating ALI.

## 1. Introduction

Acute lung injury (ALI) is an acute respiratory disease induced by pulmonary factors (such as lung trauma and pneumonia) or extrapulmonary factors (such as sepsis and transfusion overload) and is characterized by acute hypoxic respiratory failure accompanied by pathological manifestations, including alveolar epithelial and capillary endothelial injury, inflammatory cell infiltration, increased capillary permeability, and pulmonary effusion [1]. If not promptly and effectively implemented, ALI can progress to acute respiratory distress syndrome (ARDS) and even lead to multiple organ failure [2]. Previous studies have highlighted the critical role of proinflammatory macrophage polarization in the pathogenesis of ALI [3]. The excessive accumulation of proinflammatory cytokines and proteases secreted by M1‐like macrophages can exacerbate lung tissue damage [4]. Research has shown that inhibiting the M1 polarization of macrophages markedly ameliorates LPS‐induced ALI [5]. Therefore, exploring strategies to inhibit the M1 polarization of macrophages is highly important for the treatment of ALI.

Mesenchymal stem cells (MSCs) have been shown to regulate immune responses and inflammation and play important roles in respiratory diseases; for example, dental pulp stem cells have been proposed as an immunomodulatory and regenerative cell source for the lung injury associated with coronavirus disease 2019, and MSCs can be a potential therapeutic approach for ALI [6]. Qian et al. [7] demonstrated that the injection of bone marrow MSCs (BMSCs) significantly alleviated alveolar neutrophil infiltration, reduced inflammation, and improved pathological lung damage in LPS‐induced ALI mice. However, unresolved issues remain, such as safety, standardized assays for cell potency, and practical clinical applications [8]. In contrast, exosomes (Exos) derived from MSCs present multiple advantages, including low immunogenicity, the absence of tumorigenicity, and low storage costs [9]. Previous studies have shown that BMSC‐derived Exos (BMSC‐Exos) can inhibit lung epithelial apoptosis, restore alveolar epithelial barrier function, and ameliorate sulfur mustard‐induced ALI [10]. Notably, Xu et al. [11] reported that BMSC‐Exos promote macrophage M2 polarization, reduce inflammation, and alleviate pathological damage in rats with LPS‐induced ALI. This study further explored the regulatory role and molecular mechanisms of BMSC‐Exos in macrophage polarization in ALI.

The PI3K/AKT signaling pathway is a core survival signaling axis within cells and plays a crucial role in regulating cell survival, proliferation, and metabolism [12, 13]. Wang et al. [14] conducted bioinformatics analysis on Qingfei Yin (a traditional Chinese medicine formula used to treat ALI) and revealed that PI3K/AKT is a key pathway enriched by Qingfei Yin and may regulate inflammation, apoptosis, and oxidative stress to ameliorate ALI. Additionally, other studies have shown that grape seed proanthocyanidins regulate macrophage polarization from M1 to M2a through the PI3K/AKT pathway, improving LPS‐induced ALI [15]. However, the relationship between BMSC‐Exos and PI3K/AKT signaling during macrophage polarization in ALI remains largely unclear. Here, we established in vivo and in vitro models of ALI using LPS‐treated mice and RAW264.7 macrophages.

## 2. Materials and Methods

### 2.1. Extraction and Characterization of BMSC‐Exos

BMSCs were cultured until they reached 80% confluence. The medium was replaced with Exos‐free complete medium (C0925S; Beyotime, China), and the cell medium was collected every 48 h. Then, all the collected medium was cleared by sequential centrifugation at 300 × *g* for 10 min, 2000 × *g* for 10 min, and 10,000 × *g* for 30 min at 4°C and passed through a 0.22‐µm filter. Exos were subsequently isolated using an Exos isolation kit (4478359; Thermo Fisher Scientific, USA) according to the manufacturer’s instructions and resuspended in PBS. The particle size distribution of the preparations was determined by nanoparticle tracking analysis (NTA) using a NanoSight NS300 instrument. Additionally, the Exos were placed on 300‐mesh copper grids, stained with 2% uranyl acetate, and observed for morphology using transmission electron microscopy (TEM). Furthermore, the expression levels of the Exos markers alix, TSG101, and CD63 and the negative markers calnexin and GM130 were detected by western blotting.

### 2.2. Cell Culture and Treatment

Human BMSCs (CP‐H166) were purchased from Wuhan Procell Life Technology Co., Ltd. and cultured in a MSC‐specific basal medium (CM‐H166; Procell, China) produced by the company. Mouse monocyte‐macrophage RAW264.7 cells were purchased from the Shanghai Cell Bank of the Chinese Academy of Sciences (SCSP‐5036; Shanghai, China), cultured in DMEM (11965084; Gibco, USA) supplemented with 10% fetal bovine serum (FBS; 10099141C; Gibco, USA) and 100 U/mL penicillin‒streptomycin (15140122; Gibco, USA), and maintained in a humidified incubator at 37°C with 5% CO_2_. A cell injury model was constructed by stimulating RAW264.7 cells with 1 μg/mL LPS (437627; Sigma, USA) for 24 h [16].

To investigate the effect of BMSC‐Exos on macrophage polarization, after LPS stimulation, the cells were treated with 10 μg/mL BMSC‐Exos for 24 h [17]. Additionally, 10 μM PI3K/AKT pathway activator 740 Y‐P (SML4269; Sigma, USA) was used to pretreat LPS‐induced macrophages for 1 h, followed by the addition of BMSC‐Exos for another 24 h to verify whether BMSC‐Exos regulate macrophage polarization through this pathway. For the LPS + inhibitor‐BMSC‐Exos group, the NC inhibitor or miR‐451a inhibitor was first transfected into BMSCs for 48 h, and after the transfection efficiency was confirmed using RT‐qPCR, the Exos were isolated from the transfected cells and cocultured with LPS‐stimulated macrophages for 24 h.

To construct a stable TLR4‐overexpressing (OE‐TLR4) RAW264.7 cell line, the cells were seeded in 6‐well plates at a density of 5 × 10^4^/mL. When the cell confluence reached 30%, lentivirus containing OE‐TLR4 (titer: 1 × 10^8^ TU/mL; MOI = 100) was added to infect the cells for 14 h. Subsequently, the medium was replaced with a complete medium for 72 h, after which the stably transfected cells were screened using 4 μg/mL puromycin (ST551; Beyotime, China). Western blotting was used to evaluate the overexpression efficiency of TLR4. OE‐NC was used as a matched negative control. In addition, the NC mimic, or miR‐451a mimic, was transfected into RAW264.7 cells using Lipofectamine 3000 (L3000150; Invitrogen, USA) according to the manufacturer’s instructions. RT‐qPCR was used to detect the overexpression efficiency of miR‐451a. miR‐451a inhibitor/mimic and OE‐TLR4 vectors were provided by GenePharma (Shanghai, China).

### 2.3. Construction of the ALI Mouse Model

Thirty male C57BL/6J mice (22 ± 2 g, 8 weeks) were purchased from SJA Laboratory Animal Co., Ltd. (Hunan, China). The mice were allowed free access to food and water and were adaptively fed for 1 week. The mice were subsequently randomly divided into Control, LPS, LPS + BMSC‐Exos, LPS + inhibitor‐BMSC‐Exos, and LPS + BMSC‐Exos+OE‐TLR4 groups, with five mice in each group for statistical analysis. Mice that died within 24 h postsurgery were excluded from the analysis. An ALI model was constructed via the tracheal injection of LPS. Briefly, the mice were anesthetized, and their tracheas were exposed. A microsyringe was used to slowly inject the LPS solution (from *Escherichia coli* O111:B4; LPS25; Sigma‒Aldrich, USA) into the lungs at a dose of 5 mg/kg from the distal end of the trachea. The successful establishment of the model was confirmed by the presence of moist rales in the lungs, and the incision was sutured. The control group mice were injected with saline. Four h after LPS injection, 200 μL of PBS containing 10 μg/mL BMSC‐Exos was administered to the mice via tail vein injection (total dose of 2 µg exosomal protein per animal). The activity of the mice was observed every 4 h [18]. Additionally, to investigate whether the effect of BMSC‐Exos depends on the expression of miR‐451a, BMSC‐Exos in which miR‐451a was knocked down were injected into the tail vein, after which the corresponding indicators were measured. Furthermore, to verify whether BMSC‐Exos exert their effects by regulating TLR4, BMSC‐Exos and the OE‐TLR4‐lentiviral vector (5 × 10^7^ pfu per mouse) were administered via tail vein injection. After 24 h of LPS induction, the mice were euthanized by intraperitoneal injection of pentobarbital sodium, and bronchoalveolar lavage fluid (BALF) and lung tissue were immediately collected. The left lung tissues were immediately weighed (left wet weight) and then dried in an 80°C oven for 48 h to measure the dry weight, after which the wet‐to‐dry mass ratio of the left lungs was calculated. The right lung tissues were used for molecular and pathological tests. A schematic timeline of the experimental procedures is shown in Figure S1. In this study, to ensure the rigor and accuracy of the research, the animals were randomly assigned to groups. The experimental results were analyzed by researchers who were blinded to the group allocations. Furthermore, no statistical methods were used to determine the number of mice in each group prior to the experiment. The sample size for each group was determined based on our previous research using this model.

### 2.4. BALF Assessment

After the experiment concluded, a small incision was made below the tracheal cricoid cartilage, and a 23G blunt needle was inserted and secured. Precooled PBS was used for sequential lavage, which was performed three times, 0.5 mL each time, to obtain BALF samples (recovery rate >80%). The samples were subsequently centrifuged at 1500 rpm for 5 min at 4°C. The supernatant was collected, and total protein levels were analyzed using a BCA protein assay kit (P0009; Beyotime, China). The pelleted cells were resuspended, and the total cell count in the BALF was assessed using a hemocytometer.

### 2.5. ELISA

The levels of IL‐1β, IL‐6, and TNF‐α in the BALF and cell supernatants from each group were measured using mouse IL‐1β kits (SEKM‐0002; Solarbio, China), mouse IL‐6 kits (SEKM‐0007; Solarbio, China), and mouse TNF‐α kits (SEKM‐0034; Solarbio, China), respectively. Following the kit instructions, standards, and test samples, biotinylated antibodies, enzyme‐conjugate working solutions, and chromogenic substrates were sequentially added, incubated, and washed. After drying, a stop solution was added, and the OD values at a wavelength of 450 nm were detected using a microplate reader (ELX‐800, Bio‐Tek, USA).

### 2.6. HE Staining

Pathological changes in the lung tissues of the mice in each group were detected using a HE staining kit (C0105S; Beyotime, China). After the experiment, the lung tissues of the mice in each group were fixed in 4% paraformaldehyde (P1110; Solarbio, China), dehydrated in ethanol, and embedded in paraffin to be cut into 5‐μm thin sections. Before HE staining, the sections were deparaffinized with xylene, rinsed with gradient ethanol, stained with hematoxylin staining solution for 5 min, differentiated with differentiation solution for a few seconds, and counterstained with eosin for 30 s. After that, the sections were dehydrated, cleared, mounted, and then observed. The severity of ALI was evaluated based on the previously described lung tissue pathological injury scoring method [18]. Scoring was performed by investigators who were blinded to the group allocation.

### 2.7. Immunofluorescence

After treatment, the RAW264.7 cells were fixed with 4% paraformaldehyde for 15 min and then permeabilized with an immunostaining permeabilization solution (P0096; Beyotime, China) for 10 min. The cells were subsequently blocked with 5% BSA (SW3015; Solarbio, China) at room temperature for 1 h. Primary antibody against iNOS (1:500; ab178945; Abcam, UK) was added, and the samples were incubated overnight at 4°C. The following day, a goat anti‐rabbit IgG H&L (Alexa Fluor 647) secondary antibody (1:1000; ab150083; Abcam, UK) was added and incubated in the dark for 1 h. After the cells were washed with PBS, the nuclei were stained with DAPI (ab285390; Abcam, UK) for 10 min. The slides were mounted with an antifluorescence quenching mounting medium (P0126; Beyotime, China) and observed under a fluorescence microscope. For immunofluorescence double staining of lung tissue sections, antibodies against the macrophage marker F4/80 (1:200; ab90247; Abcam, UK) and the M1 polarization marker iNOS (1:200; ab178945; Abcam, UK) were used. Additionally, to confirm the uptake of Exos by macrophages, PKH‐26 (C2071S; Beyotime, China) was used to label Exos for observation. Briefly, 50 μL of the extracted Exos was incubated with PKH26 working solution (2 µL of PKH26 + 1.25 µL of enhancer + 46.75 µL of assay buffer) at 37°C in the dark for 5 min, followed by incubation at 4°C in the dark for 15 min. The mixture was then centrifuged to remove the free dye and resuspended in PBS. The RAW264.7 cell culture medium was replaced with penicillin‒streptomycin‐free medium, and PKH26‐labeled Exos (10 µg/mL) were added and incubated at 37°C for 4 h. After being washed with PBS, the cells were fixed with 4% paraformaldehyde, stained with DAPI, and observed under a fluorescence microscope.

### 2.8. Western Blot

Proteins were extracted from lung tissues, cells, and Exos in RIPA buffer (P0038; Beyotime, China) containing protease and phosphatase inhibitors (P1045; Beyotime, China), and the protein concentration was measured using a BCA kit (23225; Thermo Fisher Scientific, USA). The proteins were separated by SDS‐PAGE and transferred onto PVDF membranes (FFP24; Beyotime, China). The membranes were then blocked with 5% BSA for 1 h, followed by overnight incubation with primary antibodies at 4°C. The primary antibodies used included CD63 (1:1000; ab315108; Abcam, UK), alix (1:1000; ab275377; Abcam, UK), TSG101 (1:1000; ab125011; Abcam, UK), calnexin (1:1000; ab22595; Abcam, UK), GM130 (1:1000; ab52649; Abcam, UK), iNOS (1:1000; ab178945; Abcam, UK), CD80 (1:2000; 66406‐1‐Ig; Proteintech, China), CD86 (1:2000; 13395‐1‐AP; Proteintech, China), p‐PI3K (1:1000; PA5‐17387; Thermo Fisher Scientific, USA), PI3K (1:1000; #4257; CST, USA), p‐AKT (1:1000; #9271; CST, USA), AKT (1:1000; #9272; CST, USA), TLR4 (1:200; 48‐2300; Thermo Fisher Scientific, USA), and β‐actin (1:1000; ab8227; Abcam, UK). After the membranes were washed three times with TBST, they were incubated with HRP‐conjugated goat anti‐rabbit and goat anti‐mouse secondary antibodies (1:2000; ab205718/ab6789; Abcam, UK) at room temperature for 1 h. After the incubation, visualization was performed using a kit (P0018S; Beyotime, China).

### 2.9. RT‐qPCR

Total RNA was extracted from the lung tissues of mice in each group or from RAW264.7 cells using the TRIzol reagent (R0016; Beyotime, China). RT‐PCR was performed using a one‐step real‐time fluorescence quantitative kit (T2210; Solarbio, China) according to the manufacturer’s instructions. U6 was used as the internal control for miR‐451a, and β‐actin was used as the internal control for the mRNA targets. The data were analyzed using the 2^−ΔΔCt^ method. The primer sequences are shown in Table 1.

**Table 1:** Primer sequences.

Genes	Primer sequences (5′–3′)
iNOS (mouse)	F: CAACAATACAAGATGACCCTAAGAG
	R: ATTCTGGAACATTCTGTGCTG
CD80 (mouse)	F: GATTCGGCGCAGTAATAACAG
	R: TTCTTCTGAAACAGCTTCTGTG
CD86 (mouse)	F: GAAAGCACATAGTGTATCTATACGG
	R: CATTACAAGCATGAGCCCTG
miR‐451a (mouse)	F: CGCGAAACCGTTACCATTAC
	R: AGTGCAGGGTCCGAGGTATT
miR‐451a (human)	F: CGCGAAACCGTTACCATTAC
	R: AGTGCAGGGTCCGAGGTATT
β‐Actin (mouse)	F: GTGACGTTGACATCCGTAAAGA
	R: GCCGGACTCATCGTACTCC
U6 (mouse)	F: CTCGCTTCGGCAGCACA
	R: AACGCTTCACGAATTTGCGT
U6 (human)	F: CTCGCTTCGGCAGCACA
	R: AACGCTTCACGAATTTGCGT

### 2.10. Dual‐Luciferase Reporter Gene Assay

The binding sites between miR‐451a and TLR4 were predicted via online tools. The 3^′^—UTRs of wild‐type TLR4 (TLR4‐WT) and its mutant (TLR4‐MUT) were cloned and inserted into the pmirGLO dual‐luciferase vector (GeneChem, China). These vectors, along with the miR‐451a mimic and control mimic, were cotransfected into cells using the Lipofectamine 3000 reagent (L3000150; Invitrogen, USA). After 48 h, the cells were collected and lysed, and the luciferase activity was measured using a dual‐luciferase reporter gene assay kit (D0011; Solarbio, China).

### 2.11. Bioinformatics Analysis

Two publicly available datasets deposited in the NCBI Gene Expression Omnibus (GEO) were reanalyzed. GSE147138 (*M. musculus*; Illumina HiSeq 4000, GPL21103; small RNA sequencing of lung tissue; *n* = 15) profiles microRNA expression in the lungs of wild‐type and R213G extracellular superoxide dismutase mice 7 days after a single intratracheal administration of bleomycin (100 µL at 1 U/mL) or PBS and comprises four groups: wild‐type + PBS (*n* = 3), wild‐type + bleomycin (*n* = 4), R213G + PBS (*n* = 4) and R213G + bleomycin (*n* = 4). GSE229262 (*Mus musculus*; Agilent‐085983 Arraystar Mouse lncRNA V4 microarray, GPL26962; long noncoding RNA and mRNA expression profiling of lung tissue; *n* = 9) profiles the lungs of mice fed a normal diet (*n* = 3) or a high‐fat diet (*n* = 3) and of high‐fat diet‐fed mice given LPS as a model of obesity‐related ALI (*n* = 3). Differential expression was defined as |log_2_ fold change| > 1 and *p* value < 0.05.

### 2.12. Statistical Analysis

All the data were analyzed using GraphPad Prism 8.0 software and are expressed as the mean ± standard deviation (mean ± SD). For the in vivo experiments, the experimental unit was the individual animal (*n* = 5 mice per group); for the in vitro experiments, it was the independent biological replicate (*n* = 3 independent biological replicates). Normality was assessed with the Shapiro–Wilk test, and homogeneity of variance was assessed with the Brown–Forsythe test. All the data satisfy these assumptions. Comparisons between two groups were conducted using independent sample *t* tests, and comparisons among multiple groups were analyzed by one‐way ANOVA with Fisher’s LSD post hoc test. *p* < 0.05 was considered to indicate statistical significance.

## 3. Results

### 3.1. BMSC‐Exos Inhibited ALI in Mice

BMSC‐Exos were isolated from BMSC medium as described in Section 2.1 and characterized by TEM, NTA, and western blotting. TEM and NTA revealed that the Exos derived from BMSCs exhibited cup‐shaped vesicles with a typical concave morphology, with diameters ranging between 30 and 150 nm (Figure 1A,B). Western blot analysis showed that the Exos markers CD63, alix, and TSG101 were enriched in BMSC‐Exos, whereas the negative markers calnexin and GM130 were depleted in BMSC‐Exos (Figure 1C), indicating successful Exos extraction. The protective effects of BMSC‐Exos against LPS‐induced ALI in mice were subsequently evaluated. Compared with those in the control group, the alveolar structure in the mice in the LPS group was disrupted, with alveolar wall edema, thickening, reduced alveolar space, and pronounced inflammatory cell infiltration, whereas lung tissue pathological damage was significantly alleviated in the BMSC‐Exos group compared with that in the LPS group (Figure 1D). Additionally, compared with the LPS group, the LPS + BMSC‐Exos group presented significant decreases in the lung wet/dry weight ratio, BALF protein concentration, and BALF total cell count (Figure 1E). ELISA revealed that LPS induction significantly increased the levels of the proinflammatory factors IL‐1β, IL‐6, and TNF‐α in the BALF, whereas treatment with BMSC‐Exos treatment markedly reduced the levels of these factors (Figure 1F). These results indicate that BMSC‐Exos can inhibit ALI in mice.

Figure 1BMSC‐Exos inhibit ALI in mice. (A) Morphology of Exos under TEM. (B) Exos size distribution measured by NTA. (C) Western blot detection of CD63, alix, TSG101, calnexin, and GM130 expression. (D) HE staining showing pathological changes in the lung tissues of each group of mice; scale bar: 25 μm. (E) Lung wet/dry weight ratio, BALF protein concentration, and total cell count in each group of mice. (F) ELISA detection of IL‐1β, IL‐6, and TNF‐α levels in the BALF. The data are expressed as the mean ± SD; *n* = 5 mice per group for (D–F), and *n* = 3 independent exosome preparations for (A–C).  ^∗∗∗^ *p* < 0.001.

**Figure:**
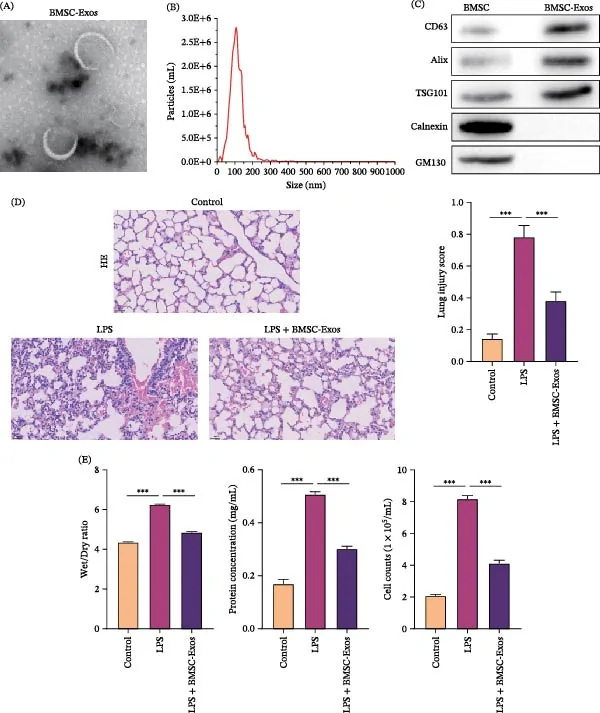

**Figure:**
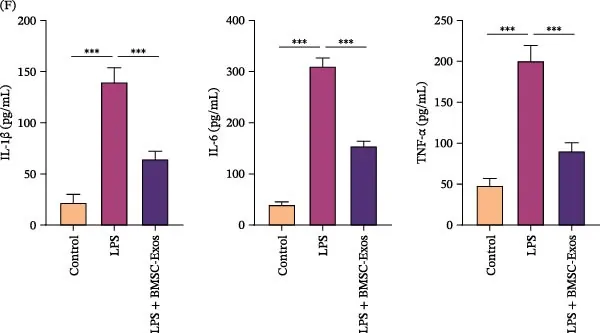

### 3.2. BMSC‐Exos Inhibited M1 Macrophage Polarization

Previous studies have shown that the inflammatory response mediated by M1 macrophage polarization plays a significant role in ALI [19]. To investigate the effect of BMSC‐Exos on M1 macrophage polarization, Exos isolated from BMSCs were cocultured with LPS‐induced macrophages. Compared with those in the control group, the expression levels of the M1 macrophage markers iNOS, CD80, and CD86 in the lung tissues of ALI mice significantly increased, whereas treatment with BMSC‐Exos treatment markedly reduced the expression of these proteins (Figure 2A). To confirm that the macrophages take up the BMSC‐Exos, the Exos were labeled with PKH‐26. Staining revealed a significant increase in red fluorescence within the macrophages, indicating that the macrophages could take up the BMSC‐Exos (Figure 2B). In vitro experiments demonstrated that LPS stimulation significantly increased the expression of iNOS, CD80, and CD86 in RAW264.7 cells, which was notably reduced after treatment with BMSC‐Exos (Figure 2C,D). Additionally, LPS treatment significantly increased the levels of IL‐1β, IL‐6, and TNF‐α, whereas treatment with BMSC‐Exos decreased the levels of these cytokines (Figure 2E). These results suggest that BMSC‐Exos can inhibit M1 macrophage polarization in ALI, thereby alleviating the inflammatory response.

**Figure 2:**
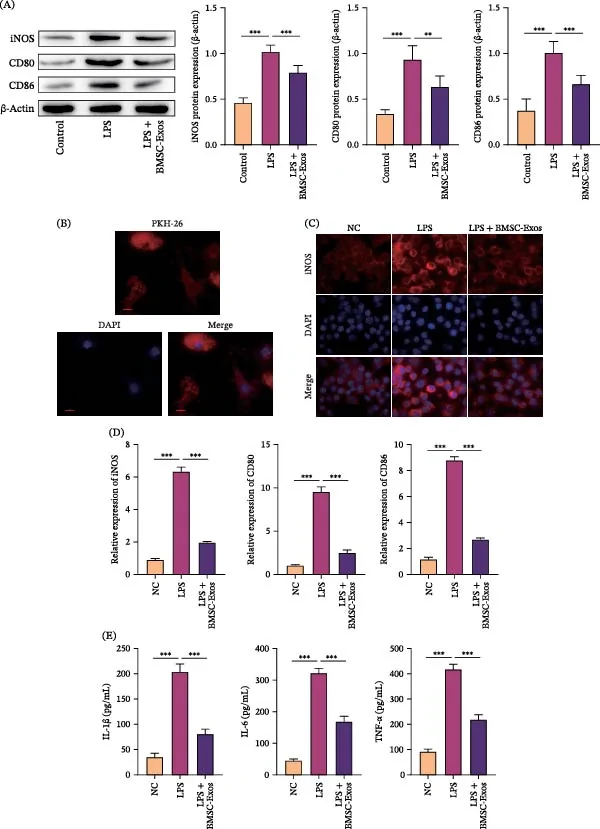
BMSC‐Exos inhibit M1 macrophage polarization. (A) Western blot detection of iNOS, CD80, and CD86 expression in lung tissues from mice in each group. (B) Detection of macrophage uptake of PKH‐26‐labeled Exos; scale bar: 10 μm. (C) Immunofluorescence detection of iNOS expression in cells from each group; scale bar: 10 μm. (D) RT‐qPCR detection of iNOS, CD80, and CD86 mRNA expression in cells. (E) ELISA detection of IL‐1β, IL‐6, and TNF‐α levels in cell supernatants from each group. The data are expressed as the mean ± SD; *n* = 5 mice per group (A), and *n* = 3 independent biological replicates (B–E).  ^∗∗^ *p* < 0.01,  ^∗∗∗^ *p* < 0.001.

### 3.3. BMSC‐Exos Inhibited Macrophage M1 Polarization by Suppressing the Activity of the PI3K/AKT Signaling Pathway

Previous research has indicated that the PI3K/AKT signaling pathway can promote M1 macrophage polarization [20]. To investigate whether BMSC‐Exos exert their effects through this pathway, the levels of PI3K/AKT pathway proteins were measured in vivo and in vitro. Compared with the control treatment, LPS treatment markedly increased the p‐PI3K/PI3K and p‐AKT/AKT ratios, indicating pathway activation. Treatment with BMSC‐Exos significantly reduced the p‐PI3K/PI3K and p‐AKT/AKT ratios (Figure 3A). To further verify whether BMSC‐Exos regulate macrophage M1 polarization by inhibiting the PI3K/AKT signaling pathway, the pathway activator 740 Y‐P was added to the cells. Compared with the LPS + BMSC‐Exos group, the 740 Y‐P group presented significantly increased p‐PI3K/PI3K and p‐AKT/AKT ratios, indicating the activation of the PI3K/AKT pathway (Figure 3B). Additionally, 740 Y‐P weakened the inhibitory effect of BMSC‐Exos on the expression of M1 macrophage markers, significantly increasing iNOS, CD80, and CD86 expression. Immunofluorescence staining likewise showed that 740Y‐P increased the iNOS expression in the LPS + BMSC‐Exos group (Figure 3C,D). Furthermore, compared with the LPS + BMSC‐Exos group, the 740 Y‐P treatment group exhibited significantly increased levels of IL‐1β, IL‐6, and TNF‐α (Figure 3E). These results indicate that BMSC‐Exos inhibit macrophage M1 polarization by suppressing the activity of the PI3K/AKT signaling pathway.

Figure 3BMSC‐Exos inhibit macrophage M1 polarization by suppressing the activity of the PI3K/AKT signaling pathway. (A) Western blot analysis of p‐PI3K, PI3K, p‐AKT, and AKT expression in lung tissues of mice. (B) Western blot analysis of p‐PI3K, PI3K, p‐AKT, and AKT expression in RAW264.7 cells. (C) RT‐qPCR detection of iNOS, CD80, and CD86 expression in cells from each group. (D) Immunofluorescence detection of iNOS expression in RAW264.7 cells; scale bar: 10 μm. (E) ELISA measurement of IL‐1β, IL‐6, and TNF‐α levels in cell supernatants. The data are expressed as the mean ± SD; *n* = 5 mice per group (A), and *n* = 3 independent biological replicates (B–E).  ^∗^ *p* < 0.05,  ^∗∗^ *p* < 0.01,  ^∗∗∗^ *p* < 0.001.

**Figure:**
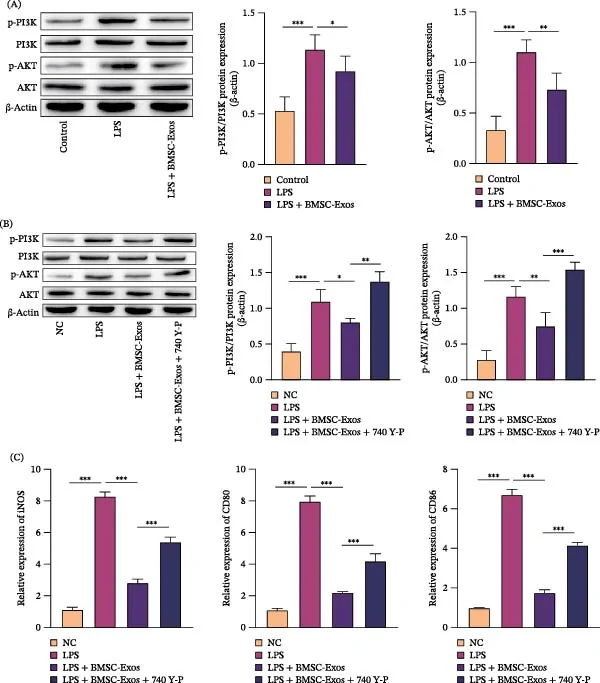

**Figure:**
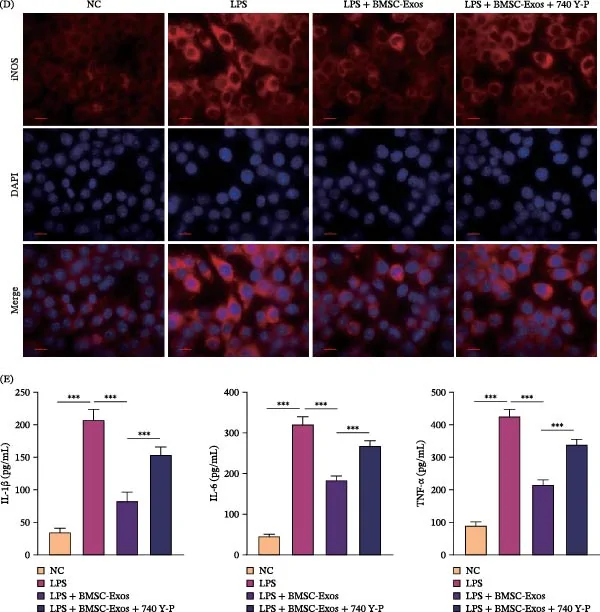

### 3.4. BMSC‐Exos‐Derived miR‐451a Inhibited Macrophage M1 Polarization by Suppressing the PI3K/AKT Signaling Pathway

Research has shown that Exos can deliver miRNAs to exert functional regulatory effects [21]. To explore the key miRNAs through which BMSC‐Exos exert their effects, the ALI miRNA dataset GSE147138 was cross‐referenced with an exosomal miRNA list, which revealed that the expression of miR‐451a was markedly decreased in ALI and substantially enriched in BMSC‐Exos (Figure 4A,B). RT‐qPCR confirmed these results as the expression of miR‐451a was significantly lower in the LPS group than in the control/NC group (Figure 4C,D). Additionally, compared with the BMSC group, the BMSC‐Exos group presented significantly higher miR‐451a levels (Figure 4E). These findings suggest that miR‐451a may be a critical molecule that mediates the effects of BMSC‐Exos. Therefore, BMSCs were transfected with a miR‐451a inhibitor, and the transfection efficiency was verified by RT‐qPCR (Figure 4F). Exos isolated from the transfected cells were subsequently cocultured with LPS‐stimulated macrophages. The RT‐qPCR results revealed that compared with the LPS group, the BMSC‐Exos group had significantly increased miR‐451a expression, whereas the inhibitor‐BMSC‐Exos group had further reduced expression (Figure 4G). Western blot analysis revealed that compared with treatment with LPS + BMSC‐Exos, treatment with inhibitor‐BMSC‐Exos significantly increased the p‐PI3K/PI3K and p‐AKT/AKT ratios (Figure 4H). Moreover, treatment with inhibitor‐BMSC‐Exos significantly increased the expression levels of iNOS, CD80, and CD86 (Figure 4I,J). Additionally, compared with the LPS + BMSC‐Exos group, the inhibitor‐BMSC‐Exos group had increased levels of the proinflammatory cytokines IL‐1β, IL‐6, and TNF‐α (Figure 4K). These results indicate that BMSC‐Exo‐derived miR‐451a inhibits macrophage M1 polarization by suppressing the PI3K/AKT signaling pathway.

Figure 4BMSC‐Exos‐derived miR‐451a inhibits macrophage M1 polarization by suppressing the PI3K/AKT signaling pathway. (A) Bioinformatics analysis of the differential expression of miR‐451a. (B) Venn diagram of the intersection between the ALI miRNA dataset GSE147138 and the BMSC exosome list. (C) RT‐qPCR detection of miR‐451a expression in lung tissues of mice in each group (D) RT‐qPCR detection of miR‐451a expression in RAW264.7 cells. (E) RT‐qPCR detection of miR‐451a expression in BMSCs and Exos. (F) RT‐qPCR detection of the transfection efficiency of the miR‐451a inhibitor. (G) RT‐qPCR detection of the effect of BMSC‐Exos and inhibitor‐BMSC‐Exos on miR‐451a expression. (H) Western blot detection of p‐PI3K, PI3K, p‐AKT, and AKT expression. (I) RT‐qPCR detection of iNOS, CD80, and CD86 expression. (J) Immunofluorescence detection of iNOS expression; scale bar: 10 μm. (K) ELISA detection of IL‐1β, IL‐6, and TNF‐α levels in the cell supernatants of each group. The data are expressed as the mean ± SD; *n* = 5 mice per group (C), and *n* = 3 independent biological replicates (D–K).  ^∗^ *p* < 0.05,  ^∗∗^ *p* < 0.01,  ^∗∗∗^ *p* < 0.001.

**Figure:**
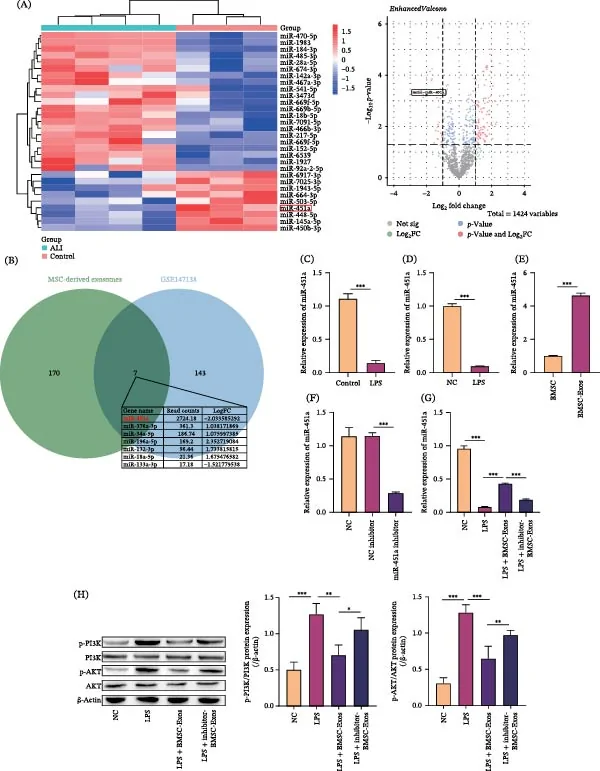

**Figure:**
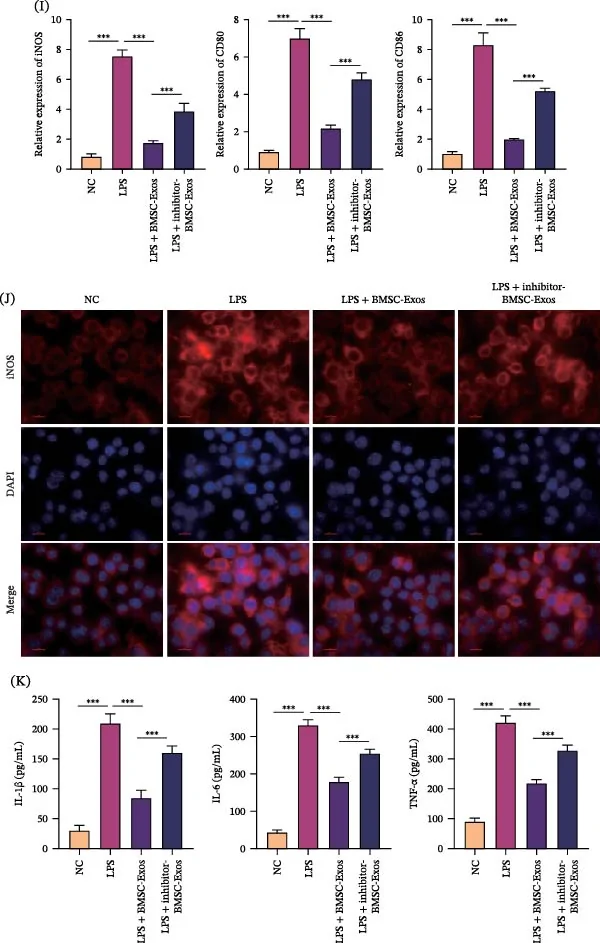

### 3.5. miR‐451a Inhibited the PI3K/AKT Signaling Pathway by Targeting TLR4

Further analysis through the starBase database revealed downstream target genes of miR‐451a, which intersected with the upregulated mRNAs listed in the ALI mRNA dataset GSE229262, resulting in eight candidate mRNAs. Among these genes, TLR4 was abnormally highly expressed in ALI and is a downstream target gene of miR‐451a (Figure 5A,B). In vivo and in vitro Western blot experiments validated these results, revealing that TLR4 expression was significantly elevated in the LPS group compared with that in the control/NC group (Figure 5C,D). The binding sites between miR‐451a and TLR4 were subsequently predicted using online tools (Figure 5E). Dual‐luciferase reporter gene assays revealed that the miR‐451a mimic significantly reduced the luciferase activity of the TLR4‐WT reporter but had no significant effect on the mutant (TLR4‐MUT) reporter (Figure 5F). Successful transfection of the miR‐451a mimic and the TLR4 overexpression vector was verified by RT‐qPCR and Western blotting, respectively (Figure 5G,5H). Western blot analysis demonstrated that compared with the NC group, the miR‐451a mimic significantly suppressed the expression of TLR4, p‐PI3K/PI3K, and p‐AKT/AKT. However, TLR4 overexpression reversed the effect of the miR‐451a mimic, partially restoring the expression of these proteins (Figure 5I). These results indicate that miR‐451a inhibits the PI3K/AKT signaling pathway by reducing the TLR4 expression.

Figure 5miR‐451a inhibits the PI3K/AKT signaling pathway by targeting TLR4. (A) Bioinformatics analysis of TLR4 expression. (B) Venn diagram of the intersection between miR‐451a downstream target genes and upregulated mRNAs in the GSE229262 dataset. (C) Western blot detection of TLR4 expression in lung tissues of mice in each group. (D) Western blot detection of TLR4 expression in cells of each group. (E) Prediction of miR‐451a and TLR4 binding sites. (F) Dual‐luciferase reporter gene assay. (G) RT‐qPCR detection of miR‐451a mimic transfection efficiency. (H) Western blot detection of OE‐TLR4 transduction efficiency. (I) Western blot detection of TLR4, p‐PI3K, PI3K, p‐AKT, and AKT expression. The data are expressed as the mean ± SD; *n* = 5 mice per group (C), and *n* = 3 independent biological replicates (D, F–I).  ^∗^ *p* < 0.05,  ^∗∗^ *p* < 0.01,  ^∗∗∗^ *p* < 0.001.

**Figure:**
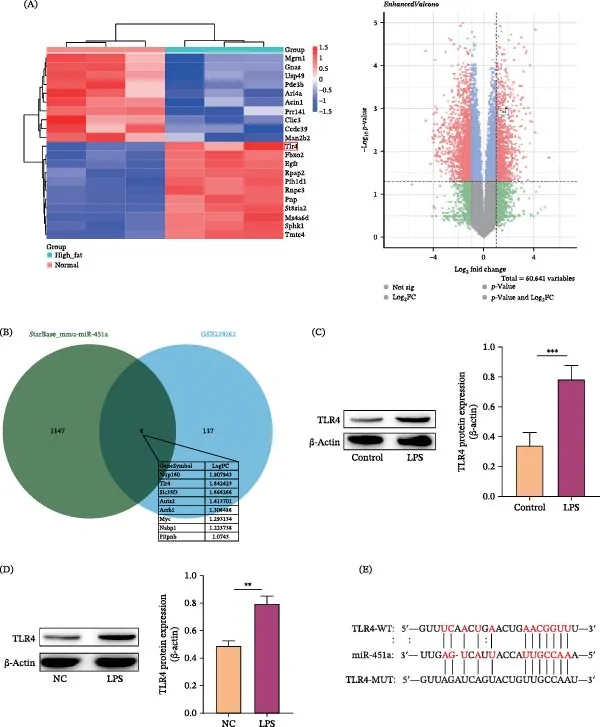

**Figure:**
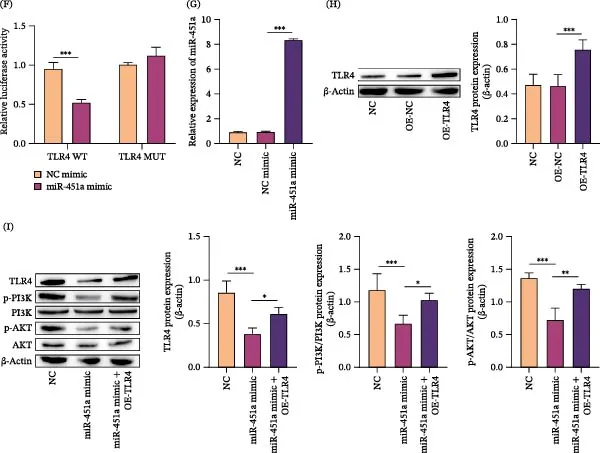

### 3.6. BMSC‐Exos‐Derived miR‐451a Inhibited ALI in Mice by Suppressing the TLR4/PI3K/AKT Signaling Pathway

Finally, the roles of miR‐451a and the TLR4/PI3K/AKT axis were verified in ALI mice. RT‐qPCR revealed that compared with LPS treatment, treatment with BMSC‐Exos significantly increased the expression of miR‐451a in lung tissue. Injection of BMSC‐Exos with miR‐451a knockdown markedly reduced miR‐451a expression, whereas TLR4 overexpression did not affect the miR‐451a expression in the LPS + BMSC‐Exos group (Figure 6A). Compared with those in the LPS group, the expression of TLR4, p‐PI3K/PI3K, and p‐AKT/AKT in the LPS + BMSC‐Exos group significantly decreased. Further injection of BMSC‐Exos with miR‐451a knockdown or TLR4 overexpression reversed the inhibitory effects of BMSC‐Exos on TLR4, p‐PI3K/PI3K, and p‐AKT/AKT expression, restoring their expression levels (Figure 6B). HE staining results demonstrated that further injection of BMSC‐Exos with miR‐451a knockdown or TLR4 overexpression attenuated the ameliorative effects of BMSC‐Exos on pathological damage to the LPS‐induced lung tissue (Figure 6C). Compared with those in the LPS + BMSC‐Exos group, the lung wet/dry weight ratio, BALF total protein concentration, and BALF total cell count were significantly increased in the groups that received the miR‐451a‐knockdown BMSC‐Exos or OE‐TLR4 (Figure 6D–F). Additionally, miR‐451a knockdown or TLR4 overexpression reversed the inhibitory effects of BMSC‐Exos on M1 macrophage markers and significantly increased the expression of iNOS, CD80, and CD86 (Figure 6G). Immunofluorescence double staining demonstrated the colocalization of iNOS and F4/80 (a macrophage marker) in lung tissue, and the expression trend of iNOS in macrophages was consistent with the Western blot results (Figure 6H). Moreover, the levels of IL‐1β, IL‐6, and TNF‐α were increased after miR‐451a knockdown or TLR4 overexpression (Figure 6I). These results indicate that BMSC‐Exos‐derived miR‐451a alleviates ALI in mice by suppressing the TLR4/PI3K/AKT signaling pathway.

Figure 6BMSC‐Exos‐derived miR‐451a alleviates mouse ALI by inhibiting TLR4/PI3K/AKT signaling. (A) RT‐qPCR detection of miR‐451a expression. (B) Western blot analysis of TLR4, p‐PI3K, PI3K, p‐AKT, and AKT expression. (C) HE staining showing lung tissue pathological changes; scale bar: 25 μm. (D) Lung wet/dry weight ratio in each group. (E) Protein concentration in the BALF of each group. (F) Total cell count in the BALF of each group. (G) Western blot detection of the expression of the M1 markers iNOS, CD80, and CD86. (H) Immunofluorescence double staining was used to detect the colocalization of iNOS and F4/80 in lung tissue; scale bar: 25 μm. (I) ELISA measurement of IL‐1β, IL‐6, and TNF‐α levels in the BALF. The data are expressed as the mean ± SD; *n* = 5 mice per group.  ^∗^ *p* < 0.05,  ^∗∗^ *p* < 0.01,  ^∗∗∗^ *p* < 0.001.

**Figure:**
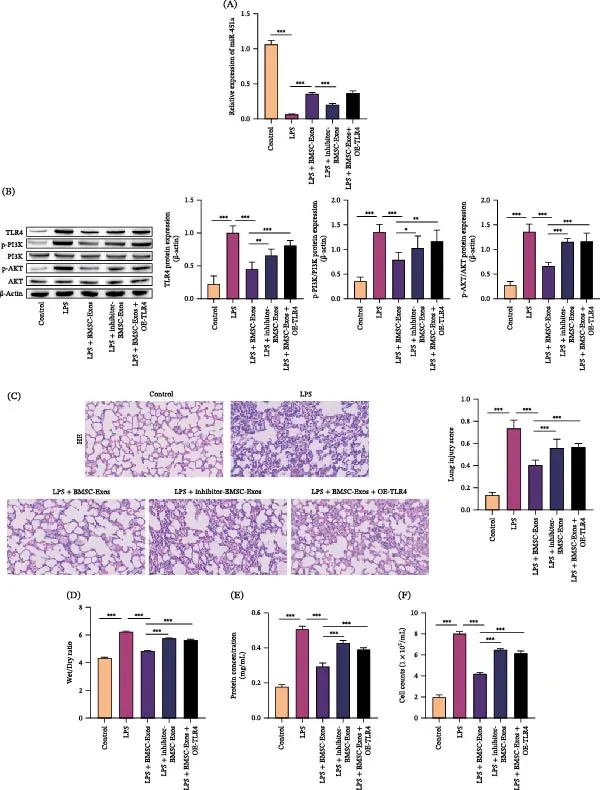

**Figure:**
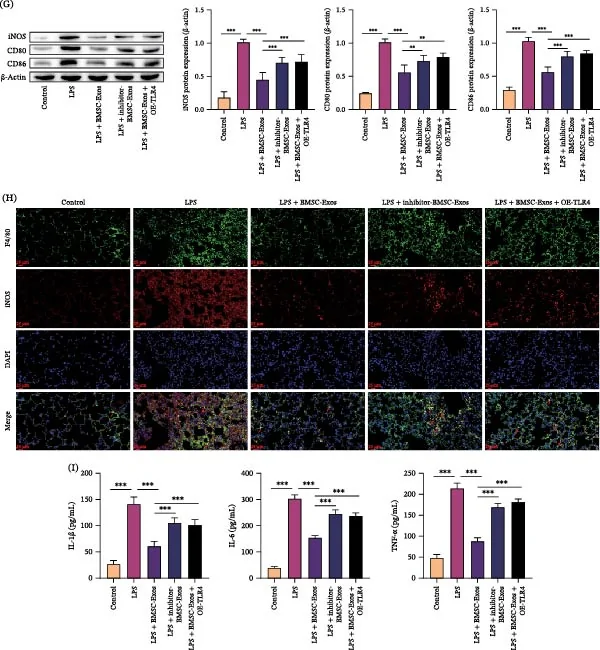

## 4. Discussion

Owing to its high incidence and mortality rates, ALI has been recognized as a medical crisis [22]. Additionally, persistent functional disabilities, such as muscle atrophy, fatigue, and polyneuropathy, impose a significant burden on patients [23]. Macrophages can polarize into different phenotypes during various stages of ALI, indicating disease progression, while excessive activation of M1 polarization can exacerbate lung tissue damage [3]. This study revealed that BMSC‐Exos can inhibit macrophage M1 polarization by suppressing the activity of the PI3K/AKT signaling pathway, thereby improving ALI in mice. Further research revealed that miR‐451a was enriched in BMSC‐Exos and suppressed the PI3K/AKT signaling pathway by reducing the expression of TLR4. However, inhibiting miR‐451a expression or overexpressing TLR4 reversed the positive effects of BMSC‐Exos. These findings provide a mechanistic basis for the targeted application of MSC‐derived Exos in ALI.

In recent years, numerous studies have highlighted the therapeutic potential of MSC‐Exos in diseases [24], as they not only possess advantages such as immunomodulatory effects, low immunogenicity, and ease of storage and administration but also overcome the safety risks and limitations of MSCs [25, 26]. Liu et al. [27] reported that MSC‐Exos can inhibit alveolar macrophage pyroptosis and alleviate inflammatory responses in ALI. Another study revealed that BMSC‐Exos can mitigate LPS‐induced ALI by alleviating autophagy disorders in alveolar macrophages [28]. These studies indicate that MSC‐Exos can ameliorate ALI through the regulation of different mechanisms. Furthermore, previous research has shown that MSC‐Exos can reshape macrophage polarization and suppress inflammation to alleviate steroid‐resistant asthma in mice [17]. Xu et al. [11] reported that BMSC‐Exos inhibit ALI inflammation by inducing macrophage polarization toward the M2 phenotype. Consistent with these findings, our study revealed that BMSC‐Exos markedly reduced the levels of the M1 polarization markers iNOS, CD80, and CD86 and the proinflammatory cytokines IL‐1β, IL‐6, and TNF‐α and alleviated lung tissue damage in ALI mice, further revealing the positive role of BMSC‐Exos in ALI.

The PI3K/AKT pathway is involved in a highly conserved signaling network in eukaryotic cells and plays a crucial role in maintaining endothelial cell function and regulating immune cell metabolism and function [29]. Numerous studies have revealed the pivotal role of this pathway in ALI. For instance, Ding et al. [30] demonstrated that the PI3K/AKT signaling pathway is the core pathway through which Gegen Qinlian decoction is used to treat ALI in mice and that blocking this pathway reversed these therapeutic effects. Zhao et al. [31] reported that *Nicandra physalodes* extracts reshaped immunometabolism by inhibiting the PI3K/AKT signaling pathway, thereby alleviating lung injury and inflammatory cell infiltration in ALI mice. Additionally, the PI3K/AKT signaling pathway is involved in regulating macrophage polarization in ALI [32]. For example, Wang et al. [33] reported that baicalin inhibits macrophage M1 polarization by suppressing the activity of the PI3K/AKT signaling pathway, thereby alleviating sepsis‐induced ALI in mice. Notably, BMSC‐Exos can modulate the activation of the PI3K/AKT signaling pathway. Wu et al. [34] demonstrated that miR‐30b‐5p carried by BMSC‐Exos delays the progression of non‐small‐cell lung cancer by inhibiting the PI3K/AKT signaling pathway. Another study indicated that SCARA5 in BMSC‐Exos suppresses the malignant behavior of colorectal cancer cells by inhibiting the PI3K/AKT signaling pathway [35]. Consistent with these observations, the results of the present study revealed that PI3K/AKT signaling is significantly activated in ALI and is accompanied by elevated M1 polarization markers. Treatment with BMSC‐Exos inhibited the expression of proteins associated with this pathway. Furthermore, 740 Y‐P treatment reversed the inhibitory effect of BMSC‐Exos on macrophage M1 polarization, indicating that BMSC‐Exos suppress macrophage M1 polarization by inhibiting the PI3K/AKT signaling pathway.

Exos are membrane‐bound small vesicles that are secreted by cells and range in diameter from 30 to 150 nm; these vesicles contain various bioactive molecules, such as proteins, lipids, mRNAs, and miRNAs, and play crucial roles in intercellular communication [36]. Current research highlights the therapeutic effects of exosomal miRNA delivery in diseases [37]. To explore the potential molecular mechanisms through which BMSC‐Exos exert their effects, we analyzed the ALI miRNA dataset GSE147138 and further compared it with the BMSC‐Exos list. The results revealed that miR‐451a expression is markedly decreased in ALI and enriched in BMSC‐Exos. miR‐451a is a highly conserved small noncoding RNA belonging to the miR‐451 family, and previous studies have focused primarily on cancer and inflammatory diseases [38]. Studies have demonstrated that ADSC‐Exos enriched with miR‐451a could promote the shift of macrophages from proinflammatory to anti‐inflammatory [39]. Our recent research revealed that exosomal miR‐451a from BMSCs suppressed the M1 polarization of macrophages through the MIF/CD74 signaling pathway to alleviate ALI [40]. In this study, we observed reduced expression of miR‐451a in ALI, and inhibition of its expression reversed the suppressive effects of BMSC‐Exos on PI3K/AKT pathway activation and M1 macrophage polarization, indicating that BMSC‐Exos‐derived miR‐451a inhibits M1 macrophage polarization by suppressing the PI3K/AKT pathway.

To identify the target through which miR‐451a blocks PI3K/AKT signaling, we retrieved genes downstream of miR‐451a from the starBase database and intersected them with the mRNAs upregulated in the ALI mRNA dataset GSE229262, which pointed to TLR4. TLR4 is a key transmembrane receptor of the innate immune system that is expressed in various cells, such as macrophages, alveolar epithelial cells, and neutrophils, and can recognize LPS to initiate immune responses [41]. Studies have shown that activation of TLR4 can mediate the activation of the PI3K/AKT signaling pathway and that inhibition of this pathway can alleviate paraquat‐induced ALI [42]. Additionally, knockout of S100A9 inhibits the activity of TLR4 and its downstream signaling axis, suppresses macrophage M1 polarization, reduces the levels of pulmonary macrophage chemokines and inflammatory cytokines, and alleviates LPS‐induced ALI [43], revealing that TLR4 can regulate the PI3K/AKT signaling pathway and macrophage polarization. Furthermore, notably, miR‐451a can regulate the expression of TLR4. Bashi et al. [44] reported that miR‐451a alleviates heme‐induced inflammation by negatively regulating TLR4. In the present study, TLR4 expression was markedly elevated in ALI and was reduced by miR‐451a overexpression. Dual‐luciferase reporter assays further confirmed the direct binding between miR‐451a and TLR4. TLR4 overexpression weakened the therapeutic effects of BMSC‐Exos, activated the PI3K/AKT pathway, upregulated the expression of M1 cell markers and inflammatory factors, and exacerbated lung injury, as validated in animal experiments. Therefore, BMSC‐Exos‐derived miR‐451a alleviated ALI by reducing TLR4 expression to inhibit PI3K/AKT signaling, thereby suppressing macrophage M1 polarization.

In this study, human BMSC‐Exos were administered to C57BL/6J mice and applied to mouse RAW264.7 macrophages; thus, the treatment was xenogeneic. Several features of the BMSC‐Exos mitigate the concern that this raises. First, the mature sequences of hsa‐miR‐451a and mmu‐miR‐451a are identical; thus, human exosomal miRNA can engage mouse transcripts without any sequence mismatch. Second, MSC‐derived vesicles carry few MHC class I or II molecules and are poorly immunogenic, and xenogeneic human MSC‐derived vesicles have repeatedly been shown to be therapeutically active and well tolerated in rodent models of lung injury [45]. However, other exosomal cargoes may not be functionally conserved across species; thus, a longer‐term study is needed to exclude delayed immune responses to repeated xenogeneic dosing.

Our study revealed the regulatory role of the BMSC‐Exos‐miR‐451a/TLR4/PI3K/AKT molecular axis in macrophage polarization during ALI. However, there are several limitations in this study. First, the mechanistic experiments were performed exclusively in the RAW264.7 cell line. Although this cell line is widely used, it is a transformed monocyte‒macrophage line whose responses may differ from those of primary cells; validation in primary alveolar macrophages or bone marrow‐derived macrophages would strengthen the physiological relevance of the findings. Second, bioinformatics predictions indicated that miR‐451a has multiple potential target genes, but this study validated only TLR4. There may be coordinated effects among multiple targets, which need further verification in the future.

## Author Contributions

Conceptualization: Jiawu Yang, Yongze Liu, and Feng Li. Methodology: Jiawu Yang, Qinglang Dai, and Yuan Liao. Data curation: Yongze Liu and Liang Zhao. Software: Zhu Tian. Validation: Yongze Liu. Formal analysis: Jiawu Yang and Yuan Liao. Investigation: Jiawu Yang and Feng Li. Resources: Feng Li. Writing – original draft preparation: Jiawu Yang and Yongze Liu. Writing – review and editing: Feng Li and Liang Zhao. Visualization: Zhu Tian. Supervision: Qinglang Dai. Funding acquisition: Feng Li.

## Funding

This research was supported by the Caiyun Postdoctoral Innovation Project and Advanced Project in Yunnan Province, China, the Scientific Research Fund Project of the Yunnan Provincial Department of Education (Grant 2024J0302), Kunming Health Science and Technology Talent Training Project Number 2025‐SW(Reserve)‐94.

## Disclosure

All the authors have read and approved the manuscript.

## Ethics Statement

All animal experiments were approved by the Experimental Animal Ethics Committee of Yunnan Zequn Biotechnology Co., Ltd., and all animal experimental protocols adhered to the ARRIVE guidelines 2.0.

## Conflicts of Interest

The authors declare no conflicts of interest.

## Supporting Information

Additional supporting information can be found online in the Supporting Information section.

## Supporting information


**Supporting Information** Figure S1: A schematic timeline of the animal experimental procedures.

## Data Availability

The data are available from the corresponding author upon reasonable request.
