# Supplementary material for: Bone Marrow Mesenchymal Stem Cell‐Derived Exosomal miR‐451a Inhibits Macrophage M1 Polarization Through the TLR4/PI3K/AKT Signaling Pathway to Alleviate Acute Lung Injury
Source: Stem Cells Int. 2026 Oct 26;2026:5080421. doi: 10.1155/sci/5080421 (PMC13710806; doi:10.1155/sci/5080421)
Supplement: Supplementary file 1 [file SCI-2026-5080421-s001.docx]

**Figure S1 Animal experimental timeline**
